# Systematic data analysis of inpatient acute geriatric wards in Austria in 2024

**DOI:** 10.1007/s10354-026-01134-x

**Published:** 2026-03-06

**Authors:** Franz Feichtner, Julian Gutheil, Peter Mrak, Peter Dovjak, Bettina Goebel, Beate Boulgaropoulos, Peter Fasching, Bernhard Iglseder

**Affiliations:** 1 HEALTH—Institute for Biomedical Research and Technologies, JOANNEUM RESEARCH, Neue Stiftingtalstrasse 2, 8010 Graz, Austria; 2 Abteilung für Innere Medizin und Akutgeriatrie/Remobilisation, Landeskrankenhaus Graz II, Standort Voitsberg, Conrad-von-Hötzendorf-Straße 31, 8570 Voitsberg, Austria; 3 Österreichische Gesellschaft für Geriatrie und Gerontologie, Hermanngasse 18/1/4, 1070 Vienna, Austria; 4 Oberösterreichische Gesundheitsholding GmbH, Salzkammergut Klinikum, Bad Ischl. Gmunden. Vöcklabruck, Miller-von-Aichholz-Str. 49, 4810 Gmunden, Austria; 5 Klinik Ottakring, 5.Medizinische Abteilung mit Endokrinologie, Rheumatologie und Akutgeriatrie mit Ambulanz, Wiener Gesundheitsverbund, Montleartstrasse 37, 1160 Vienna, Austria; 6 Universitätsklinik für Geriatrie der PMU, Uniklinikum Salzburg, Christian-Doppler-Klinik, Ignaz-Harrer-Str. 79, 5020 Salzburg, Austria

**Keywords:** Acute geriatric care, Quality assurance, Benchmarking, Systematic data analysis, Aging society, Akute geriatrische Versorgung, Qualitätssicherung, Benchmarking, Systematische Datenanalyse, Alternde Gesellschaft

## Abstract

**Background and objective:**

Inpatient acute geriatric wards provide care for older patients with multiple morbidities who require treatment due to acute events, with a focus on maintaining or restoring independence. Geriatric patients need treatment that takes into account medical, functional, psychological, and social aspects. In 2005, the Association for Quality in Geriatrics and Gerontology (QiGG) developed a uniform documentation standard for process control and quality assurance in acute geriatric inpatient care, which is used by many acute geriatric wards in Austria. Aim of this study was to conduct a reliable, systematic analysis of data collected in 2024 from acute geriatric wards in Austria, taking into account gender-specific differences, in order to provide transparency on the geriatric care situation and thereby generate a basis for scientific analysis, evidence-based planning, and managing of future care structures.

**Methods:**

Patient characteristics, therapeutic services, length of stay, and geriatric assessment parameters (self-care ability, mobility, social history, polypharmacy) were analyzed from data of 7545 patients from 15 acute geriatric wards in Austria in 2024. Data were collected using the digital Benchmarking and Reporting System CDS-BARS, and analyzed using descriptive statistics.

**Results:**

More than 50% of the patients were admitted to acute geriatric wards within 2 weeks of the acute event. Overall, almost twice as many women as men were admitted. On average, patients had around 7.6 functional impairments upon admission. Most patients remained in the acute geriatric ward for 15 to 21 days and achieved a demonstrable and quantifiable improvement in their self-care abilities and mobility during their stay. A total of 90.5% of patients who lived at home before their stay returned home afterwards.

**Conclusion:**

This study underscores the importance of acute geriatric wards in an aging society and the need for continuous development of acute geriatric structures.

## Abstract (de)

**Hintergrund und Ziel:**

In der stationären akutgeriatrischen Versorgung werden ältere, multimorbide Patient*innen betreut, die aufgrund von Akutereignissen stationär behandelt werden müssen. Der Fokus liegt auf Erhalt oder Wiederherstellung der Selbstständigkeit. Geriatrische Patient*innen benötigen eine Behandlung, die medizinische, funktionelle, psychische und soziale Aspekte berücksichtigt. Der Verein Qualität in der Geriatrie und Gerontologie (QiGG) hat einen einheitlichen Dokumentationsstandard zur Prozesssteuerung und Qualitätssicherung der stationären akutgeriatrischen Versorgung erarbeitet, der von vielen Akutgeriatrien in Österreich verwendet wird. Ziel war es, eine zuverlässige, systematische Analyse der 2024 erhobenen Daten aus Akutgeriatrien in Österreich unter Berücksichtigung geschlechtsspezifischer Unterschiede durchzuführen, um Transparenz über die geriatrische Versorgungssituation und damit eine Grundlage für wissenschaftliche Analysen, evidenzbasierte Planung und Verwaltung künftiger Versorgungsstrukturen zu schaffen.

**Methoden:**

Anhand der Daten von 7545 Patient*innen aus 15 Akutgeriatrien in Österreich aus dem Jahr 2024 wurden Patient*innencharakteristik, therapeutische Leistungen und Verweildauer, sowie Parameter des geriatrischen Assessments (Selbsthilfefähigkeit, Mobilität, Sozialanamnese, Polypharmazie) analysiert. Die Daten wurden durch das digitale Benchmarking and Reporting System CDS-BARS erhoben und mithilfe deskriptiver Statistik analysiert.

**Ergebnisse:**

Mehr als 50 % der Patient*innen wurden innerhalb von 2 Wochen nach dem Akutereignis in die Akutgeriatrie aufgenommen. Es wurden fast doppelt so viele Frauen wie Männer behandelt. Durchschnittlich lagen bei der Aufnahme rund 7,6 Funktionsstörungen vor. Die meisten Patient*innen blieben zwischen 15 und 21 Tagen in der Akutgeriatrie und erzielten während ihres Aufenthalts eine nachweisliche und quantifizierbare Verbesserung ihrer Selbsthilfefähigkeit und Mobilität. Nach dem Aufenthalt konnten 90,5 % der Patient*innen, die zuvor zu Hause gelebt hatten, wieder in ihr häusliches Umfeld zurückkehren.

**Schlussfolgerung:**

Die vorliegende Studie unterstreicht die Bedeutung der Akutgeriatrie in einer alternden Gesellschaft und die Notwendigkeit einer kontinuierlichen Weiterentwicklung akutgeriatrischer Strukturen.

## Introduction

Over the past few decades, inpatient acute geriatric wards have emerged as an essential part of inpatient care for older people in Austria, in line with the Regional Health Plan of the Federal Ministry of Health [1]. Acute geriatric wards are tailored to patients who require inpatient treatment due to acute events and who are at increased risk of losing functional abilities. The focus of the treatment lies on maintaining and restoring independence and reintegrating geriatric patients into their familiar environment. Geriatric patients require treatment and care tailored to their complex situation, which is not limited to the diagnosis and therapy of a single condition but includes medical, functional, psychological, and social aspects [2]. The core concept of Austrian acute geriatric wards is to provide specialized care for older patients with frailty and multimorbidity in the context of an acute medical event. Admissions to Austrian acute geriatric wards predominantly occur via transfers from other wards within the same hospital or from external hospitals, while additional referrals originate from general practitioners, emergency departments, nursing homes, and other healthcare providers. Austrian acute geriatric wards follow a time-limited, multidisciplinary treatment process based on Comprehensive Geriatric Assessment. After structured assessment, individualized therapeutic interventions are delivered by an interdisciplinary team including geriatricians, specially trained nursing staff, physiotherapists, occupational therapists, speech and language therapists, and social workers. Central aims are medical stabilization, functional restitution, and remobilization, with the ultimate goal of enabling patients to return safely to their homes. Internationally, this model is comparable to acute geriatric units established in English-speaking healthcare systems, which similarly combine acute medical care with structured geriatric assessment and interdisciplinary rehabilitation within a dedicated ward setting. 

In 2005, the Association for Quality in Geriatrics and Gerontology (QiGG) developed a uniform documentation standard for process control and quality assurance in inpatient acute geriatric wards, which is used by many acute geriatric wards in Austria in addition to their own documentation system. The data for this study are based on the digital benchmarking and reporting system CDS-BARS, which has been supported by QiGG and JOANNEUM RESEARCH for 20 years. The QiGG has recently been incorporated into the scientific association committed to the continuous improvement of geriatric care, namely, the Austrian Society for Geriatrics and Gerontology (ÖGGG). Previously, QiGG had coordinated quality assurance measures and enabled benchmarking across the participating acute geriatric wards.

The aim of this study was to systematically analyze data collected in 2024 from acute geriatric wards in Austria, taking into account gender-specific differences, in order to create transparency regarding geriatric care outcomes and to provide a robust basis for scientific analysis, evidence-based planning, and strategic development of future care structures.

## Materials and methods

### Data collection

The data for this study originate from the benchmarking and reporting system CDS-BARS. In 2024, all 15 participating acute geriatric wards collected standardized and quality-checked data and entered them into the central system system. The benchmarking initiative ensured that the quality of acute geriatric care was made visible, developments were documented, and an objective basis for further developing acute geriatric care was created. The participating acute geriatric wards committed themselves to a complete and high-quality data collection supported by numerous verification steps in the CDS-BARS system. For the present analyses, only complete data sets were used. As a result, sample sizes differed between individual analyses.

The data collection covered patient characteristics such as demographics, social history and care situation, admission diagnoses, referral, functional impairments, and comorbidities. In addition, therapeutic services and length of stay were recorded as well as geriatric assessments of self-care ability, mobility, social history, and polypharmacy.

The data are presented in descriptive form, both as figures and tables. Evaluations were performed using the programming language R.

### Geriatric assessment

A comprehensive geriatric assessment, which is an obligatory part of diagnostics in acute geriatric care, serves as basis for individual therapy planning. The parameters self-care ability, mobility, social history, and number of pharmacological agents were assessed [3].

#### Self-care ability

Self-care ability was assess using the Barthel Index [4]. Barthel Index score range from 0 to 100, with 0 representing complete dependence on care and 100 describing complete independence. The Barthel Index is divided into the following categories: A Barthel Index score of 0–30 indicates “largely dependent on care,” 35–80 indicates “in need of assistance,” and 85–95 indicates “in need of occasional assistance” [4, 5]. The difference between the discharge score and the admission score of the Barthel Index can be considered a measure of treatment effectiveness [6].

#### Mobility

Mobility was assessed using the Esslinger Transfer Scale and the Tinetti Test.

The Esslinger Transfer Scale describes the degree of assistance required when being transferred from a bed to a chair/wheelchair. The scale ranges from assistance level 0 (H0) to assistance level 4 (H4), where H0 indicates “no assistance” and H4 indicates “more than one person providing professional assistance.” Scores from H1 to H3 can be summarized as “one person assisting” [7].

The Tinetti Test assesses mobility by evaluating walking and balance abilities, with scores ranging from 0 to 28. A Tinetti Test score of 0 indicates an increased risk of falling. The values are divided into three categories: 0–19 indicates “significantly increased risk of falling,” 19–23 indicates “slightly increased risk of falling,” scores greater than or equal to 24 are assigned to the category “slightly restricted mobility,” and a score of 28 indicates very good mobility [8].

#### Social history

The social history includes the patient’s living situation, the care situation, and the social environment or family support.

#### Number of pharmacological agents

The number of the pharmacological agents was manually entered into the CDS-BARS.

## Results

### Patient characteristics

#### Demographics

In inpatient acute geriatric wards in Austria mainly older patients with multiple morbidities are treated. In the observation period from 1 January to 31 December 2024, a total of 7545 patient cases were recorded from 15 participating inpatient acute geriatric wards. There were 4953 women (65.6%) with an average age of 81.80 ± 7.99 years and 2592 men (34.4%) with an average age of 79.91 ± 8.44 years (Table 1).

**Table 1:** Description of the data set

Number of patients	7545
Number of women; age (mean ± SD)	4953; 81.8 ± 7.99
Number of men; age (mean ± SD)	2592; 79.91 ± 8.44
Number of gender unknown	0
Number of acute geriatric wards	15
Admission date of first patient	01.01.2024
Admission date of last patient	31.12.2024

#### Social history and care situation

A total of 7026 patients from 15 acute geriatric wards were included in the analysis. Of these, 54.7% had been cared by family members at home prior to admission to the acute geriatric ward, 23.8% had received professional help at home, and 23.5% had received informal care at home. A proportion of 15.2% of these patients had lived at home without external help prior to admission to the acute geriatric ward, 5.3% had 24‑hour care, 5.2% had mobile home nursing care, and 4.9% of these patients had been in a nursing home prior to admission to the acute geriatric ward.

#### Admission diagnoses

A total of 6524 patients (4267 women and 2257 men) from 13 acute geriatric wards were included in the analyses. Admission to an acute geriatric ward showed clear gender-specific patterns. Injuries and consequences of injuries were the most common admission diagnoses (ICD S00-T98: injuries, poisoning and certain other consequences of external causes) for both women (34.8%) and men (26%), followed by diseases of the musculoskeletal system and connective tissue (M00-M99) for women (18.9%), and circulatory diseases (I00-I99) for men (23.3%; Table 2).

**Table 2:** Primary admission diagnosis by ICD-10 groups [10] in women and men

ICD-10 group	Women (*n*)	Men (*n*)	Explanation
S00-T98	1486	586	Injury, poisoning, and certain other consequences of external causes
M00-M99	806	311	Diseases of the musculoskeletal system and connective tissue
I00-I99	675	525	Diseases of the circulatory system
J00-J99	232	162	Diseases of the respiratory system
R00-R99	215	128	Symptoms, signs, and abnormal clinical and laboratory findings, not elsewhere classified
Others	853	545	Others

*N* = 6524; number of acute geriatric wards = 13; number of women = 4267; number of men = 2257

The most common admission diagnoses within the specific ICD-10 groups [9] were fractures of the thigh bone (S72, men = 204, women = 541), consequences of lower limb injuries (T93, men = 194, women = 398), and osteoarthritis of the hip joint (M16, men = 98, women = 256).

#### Admission

Of all admissions to acute geriatric wards, 53.7% were made from wards within the same hospital and 25.3% from other hospitals. The remaining admissions stem from general practitioners, emergency departments, and nursing homes, among others. A total of 5488 patients from 13 acute geriatric wards were included in this analysis.

The time between the acute event and admission to the acute geriatric ward was 0–14 days in 57.6%, 15–28 days in 22.1%, and more than 28 days in 20.3% of all included patients. For this analysis, data from 2276 patients from nine acute geriatric wards were evaluated.

#### Functional disorders

**Fig. 1:**
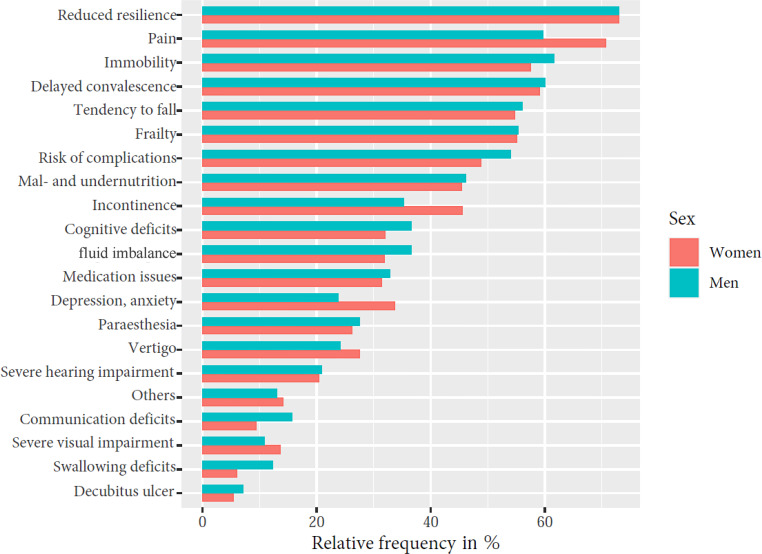
Relative frequency of functional disorders in women and men

For the analysis of functional disorders, data from 5016 patients (3295 women and 1721 men) from 13 acute geriatric wards were evaluated. Upon admission to the acute geriatric ward, both sexes had an average of 7.6 functional disorders per patient, with differences across the age cohorts. The most common functional disorders were reduced resilience, pain, immobility, delayed convalescence, and a tendency to fall (Fig. 1). These symptoms underscore the complexity of the treated patient population. There were differences between the sexes in pain (women: 70.8%, men: 59.7%), depression/anxiety (women: 33.8%, men: 23.8%), and incontinence (women: 45.6%, men: 35.3%). Women suffered more frequently from urinary incontinence, men more frequently from fecal incontinence, but we also observed differences between the sexes in swallowing deficits (women: 6%, men: 12.3%) and communication deficits (women: 9.4%, men: 15.7%; Fig. 1).

#### Comorbidities

**Fig. 2:**
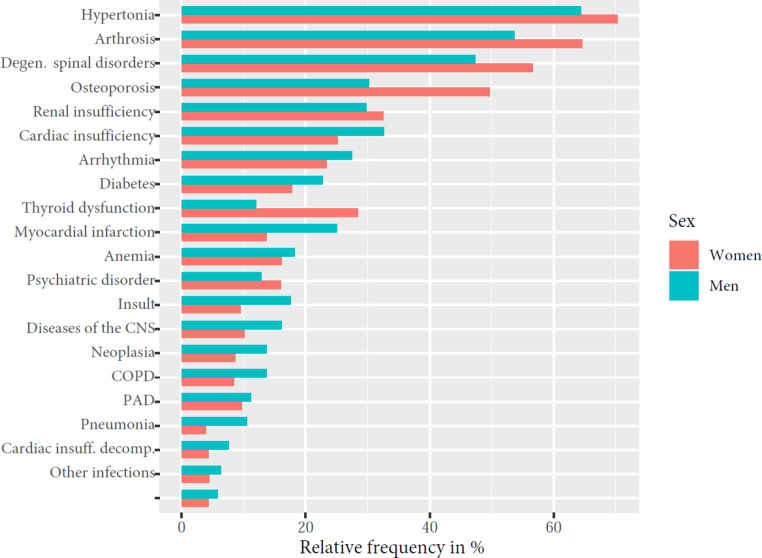
Relative frequency of most common comorbidities in women and men. *COPD* chronic obstructive pulmonary disease, *PAD* peripheral arterial disease

To analyze comorbidities, data from 1825 patients from eight acute geriatric care wards were evaluated. The multimorbidity of the patients was reflected in the large number of comorbidities documented. The most common comorbidities included arterial hypertension (68.3%), osteoarthritis (60.9%), degenerative spinal disease (53.5%), osteoporosis (43.1%), kidney failure (31.6%), and heart failure (27.7%; Fig. 2). A total of 19.5% of patients had diabetes mellitus as comorbidity.

### Therapeutic services and length of stay

A total of 3400 patients from nine acute geriatric wards were included in the analysis for therapeutic services. The following therapeutic services were provided to acute geriatric patients in descending order of frequency: physiotherapy (98.2%), ergotherapy (91.4%), activating care (88.8%), social work (63.2%), psychological care (52.2%), nutritional intervention (41.2%), brain training (10.6%), speech therapy (9.1%), continence training (8.5%), and complementary therapies (2.9%).

Data from 6913 patients from 15 acute geriatric wards were included in the analysis for the length of stay. A proportion of 4.7% of the geriatric patients spent up to 7 days in an acute geriatric ward, 12% spent between 8 and 14 days, 34.5% spent between 15 and 21 days, 28.2% spent between 21 and 28 days, and 20.5% of geriatric patients spent more than 28 days in an acute geriatric ward.

### Geriatric assessment—outcome

The patients’ self-care ability, mobility, and social history were assessed before and after their stays in the acute geriatric ward.

#### Self-care ability

**Fig. 3:**
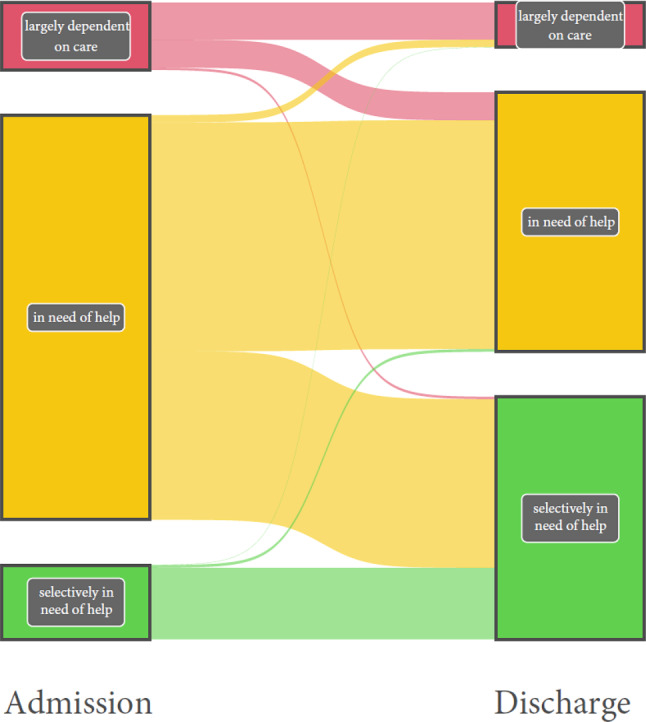
Change in self-care ability of patients during their stay in an acute geriatric ward. Left side: Barthel Index categories on admission; right side: Barthel Index categories on discharge

Self-care ability was assessed in 6557 patients from 15 acute geriatric wards. Of the patients who were “largely dependent on care” upon admission to the acute geriatric ward, 55.1% were still largely dependent on care upon discharge, 41.5% were “in need of assistance,” and 3.4% were “in need of occasional assistance.” Of those who were “in need of assistance” upon admission, 56.5% were still “in need of assistance” upon discharge, 41.7% were “in need of occasional assistance”, and the remainder (1.8%) were “largely dependent on care” upon discharge. A total of 96.4% of those who were “in need of occasional assistance” upon admission remained so upon discharge. On average, an improvement of 14.1 points was achieved on the Barthel Index, reflecting an increase in independence (Fig. 3).

In contrast to men, women were less likely to be “largely dependent on care” when admitted to acute geriatric wards (women: 12.4%, men: 16.5%). However, they were more likely to be “in need of assistance” (women: 74.4%, men: 70.9%) or “in need of occasional assistance” (women: 13.2%, men: 12.6%). Data from 7241 patients (4782 women and 2459 men) from 15 acute geriatric wards were included in this gender-specific analysis.

#### Mobility

##### Esslinger Transfer Scale

**Fig. 4:**
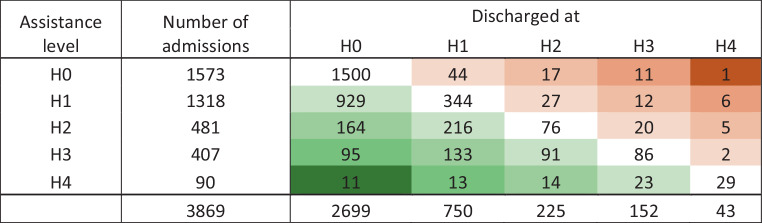
Assistance levels (H0–H4) of 3869 patients at admission and discharge according to the Esslinger Transfer Scale. Green indicates an improvement in assistance level, red a deterioration upon discharge. Uncolored fields indicate no changes in the assistance level upon discharge. The darker the color, the more pronounced the changes in assistance level

Mobility according to the Esslinger Transfer Scale was assessed in 3869 patients from 13 acute geriatric wards. Of the 1573 patients who were classified as assistance level 0 (H0) on the Esslinger Transfer Scale upon admission to the acute geriatric ward, 1500 were still in H0 upon discharge, 44 were in assistance level 1 (H1), 17 in assistance level 2 (H2), 11 in assistance level 3 (H3), and one in assistance level 4 (H4). Of the 1318 patients who were in H1 upon admission, 929 were in H0 after discharge, 344 remained in H1, 27 in H2, 12 in H3, and six in H4. Of the 481 patients who were in H2 upon admission 164 were in H0, 216 in H1, 76 remained in H2, 20 in H3, and five in H4 after discharge. Of the 407 patients who were in H3 upon admission, 95 were in H0, 133 in level H1, 91 in H2, 86 remained in H3, and two patients remained in H4 upon discharge (Fig. 4). Of the 90 patients who were in H4 when admitted to the acute geriatric ward, 11 were in H0, 13 in H1, 14 in H2, 23 in H3, and 29 in H4 after discharge.

##### Tinetti Test

**Fig. 5:**
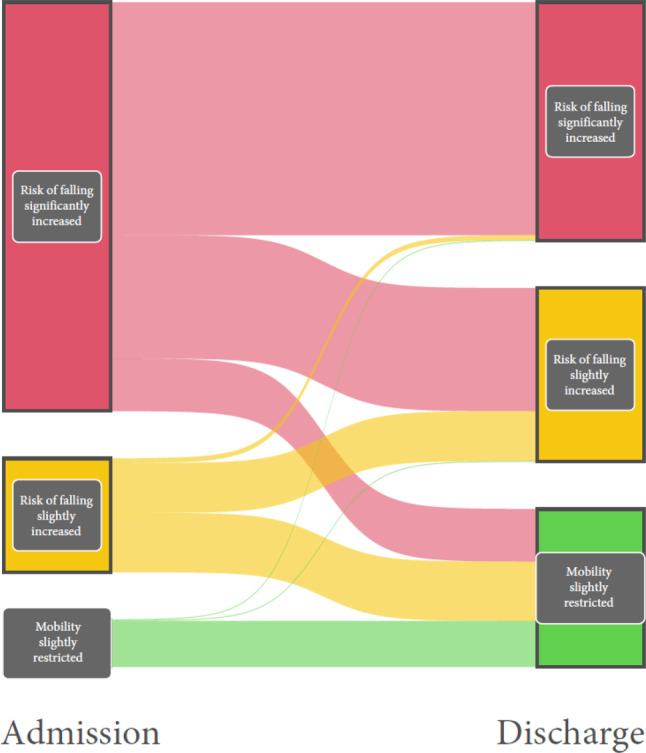
Change in mobility between admission and discharge, measured with the Tinetti Test

Data from 3414 patients from 14 acute geriatric wards were included in this analysis. Of the patients who were classified as having a “significantly increased risk of falling” according to the Tinetti Test upon admission to the acute geriatric ward, 30.1% were classified as having a “slightly increased risk of falling” upon discharge and 12.9% as having “slightly limited mobility.” Of the patients who had a “slightly increased risk of falling” upon admission, 52.1% had only “slightly limited mobility” upon discharge. Of the patients whose mobility was slightly limited upon admission, 97.2% remained so upon discharge (Fig. 5). On average, an improvement of 3.9 points was achieved in the Tinetti Test.

#### Return to home environment

Of the patients who lived at home before their stay in the acute geriatric ward, 90.5% returned home. Data from 6314 patients from 15 acute geriatric care facilities were included in this analysis.

#### Number of pharmacological agents

**Fig. 6:**
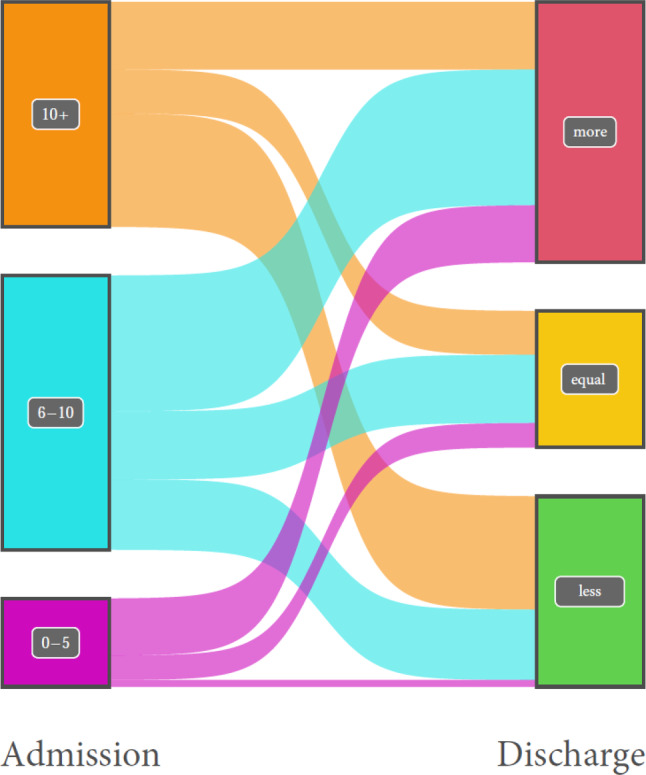
Number of pharmacological agents (≥ 10, 6–10, 0–5) at admission and change in number (more, equal, less) upon discharge

The number of pharmacological agents was evaluated in 4042 patients from 14 acute geriatric wards. Of the patients who were taking more than ten pharmacological agents upon admission to the acute geriatric ward, 50.4% had fewer pharmacological agents upon discharge. Of the patients who were taking six to ten pharmacological agents upon admission to acute geriatric ward, the number of pharmacological agents was reduced in 25.6% upon discharge and remained the same in 24.9%. Of the patients who were taking up to five pharmacological agents upon admission to acute geriatric ward, the number of pharmacological agents was reduced in 7.7% and remained the same in 27.7% (Fig. 6). On average, the number of pharmacological agents was increased by 0.4 after the stay in the acute geriatric ward compared to before.

## Discussion

The most common diagnoses leading to admission to an acute geriatric ward were injuries and consequences of injuries, with the majority attributable to age-related traumata and their consequences. In view of the demographic change in our society, this indicates a future increase in demand for age-related trauma centers.

Women and men differed with regard to the primary admission diagnosis: women more frequently presented with diseases of the musculoskeletal system and connective tissue, whereas cardiovascular diseases were more common among men. In addition, sex-specific differences were observed in functional disorders, particularly with respect to swallowing disorders. However, the prevalence of swallowing deficits in our collective was low compared to that reported previously [10]. This discrepancy might be explained by the different collectives, definitions, and assessments that were used in the past. Also, previous studies have not provided clear conclusions regarding the differences in functional disorders between men and women [11, 12].

More than half of the admissions to an acute geriatric ward (53.7%) came from other wards within the same hospital, and 25.3% of the admissions came from other hospitals. This means that almost 80% of geriatric patients were referred to an acute geriatric ward due to a need for further specialized treatment after an acute event. This reflects the care mandate of acute geriatric wards, which focuses on treating and caring for acutely ill geriatric patients. It also demonstrates the potential of these wards to relieve other acute wards while improving patient outcomes, provided patients are referred to geriatric wards appropriately and in line with available ward capacities.

In addition, the high rate of reintegration into the home environment of 90.5% of the patients observed in this study demonstrates the potential of reducing the need for long-term care.

Approximately 20% of all admissions came from general practitioners, emergency departments, or nursing homes, showing the potential for greater integration of acute geriatric wards into the primary admission process, as a geriatric approach also contributes to a better prognosis [13].

The acute geriatric care/remobilization process manual points out that timely admission after an acute event is desirable [2]. However, our data indicate that this goal is clearly not achieved: In 57.6% of cases, admission took place within 14 days of the acute event, in 22.1% within 15–28 days, and in 20.3% of cases after more than 28 days. Thus, in more than 40% of patients, admission to an acute geriatric ward did not take place within a period that meets the “timely” requirement.

Diseases are considered comorbidities when they occur alongside a specific primary disease (index disease) that represents the main disease burden. The term *multimorbidity* generally refers to two or more chronic diseases occurring simultaneously, each of which has a comparable impact on the individual’s disease burden. Differences in the incidence and prevalence of multimorbidity are substantial and depend on the population, study design, population, setting, and contextual factors. The number and type of comorbidities in the geriatric patients included were comparable to those reported in other patient cohorts of similar age [14, 15]. The high degree of multimorbidity within the present study population is reflected both in the comorbidities and the spectrum of functional disorders.

An increasing number of pharmacological agents is associated with a higher risk of related problems [16]. The high number of pharmacological agents in our data set is consistent with that reported previously [17]. Changes in the number of pharmacological agents during the stay in an acute geriatric ward varied within our patient cohort. However, it should be emphasized that the quality of pharmacological treatment cannot be assessed solely on the basis of the number of pharmacological agents adminstered [16].

In general, the risk of an increase in the number of pharmacological agents rises with the number of comorbidities. It is estimated that around 30% of people aged 65 and older take five or more pharmacological agents [18]. In the patient cohort examined in this study, the percentage was probably higher due to acute illness in the presence of existing chronic diseases. The observed slight increase in the number of pharmacological agents taken until discharge may be explained by newly diagnosed diseases in the course of the acute event.

The observed reduction in the number of prescribed pharmacological agents at discharge in our study highlights the contribution of acute geriatric care to structured prescribing. Medication adjustment represents a very complex process that requires comprehensive documentation of all current pharmacological agents, critical evaluation of their ongoing indications, and careful modification of treatment regimens, particularly in older patients who often continue taking their usual medications even though they might be “potentially inappropriate pharmacological agents.” The fact that in the present study the number of pharmacological agents was reduced at discharge in more than half of the patients who were taking more than ten pharmacological agents is remarkable, given that the patients were admitted to an acute geriatric ward due to an acute medical condition, and, therefore, an increase would have been expected.

Women tended to acieve better results in geriatric assessments than men. This finding contradicts with data from the Austrian Women’s Health Report, which indicates that although in Austria have higher live expectancy (83.7 years vs 79.4 years for men) they spend a longer period of their lives in poor to moderate health (19.3 years compared to 16.2 years in men) [19]. This finding may be attributable to the higher proportion of missing data on comorbidities in men or to the fact that the women in the patient cohort examined here were slightly younger relatve to the average female life expectancy.

The observed sex differences in functional disorders may be partly attributable to anatomical, physiological, and hormonal factors. For example, urinary incontinence is generally more prevalent in older women reflecting the higher risk of pelvic floor insufficiency associated with prior pregnancies as well as age-related declines in estrogen levels [20]. The decline of female hormones in later life also increases nociceptive sensitivity and, in combination with psychosocial factors, can lead to increased reports of pain in women [21]. The observed sex differences underscore the importance of tailored diagnostic and therapeutic approaches that adequately account for sex-related factors.

A total of 90.5% of patients who lived at home prior to the acute event were able to return home after receiving appropriate treatment in an acute geriatric ward. This high return rate and the improvement in self-care abilities and mobility underscore the effectiveness of the acute geriatric treatment concept and its importance for the quality of life of those affected.

The participating centers follow the recommendation of the “Process Handbook on Geriatric Acute Care/Remobilisation” to participate in a quality management and benchmarking system [2]. They act as part of the Austrian benchmarking initiative on professional practice in acute geriatric wards and they use the benchmarking and reporting system CDS-BARS to document care processes defined in the Austrian “Process Handbook on Geriatric Acute Care/Remobilisation” [2]. It is thus an inherent endeavor of the participating acute geriatric wards to collect complete and high-quality data. In addition, CDS-BARS incorporates multiple validation procedures to ensure the highest possible data quality. Nevertheless, missing data cannot be entirely excluded. For example, individual data may not have been collected if specific assessments could not be performed in individual patients or if certain assessments were not routinely implemented in a given acute geriatric ward. In addition, missing data may occur for variables that are not mandatory within the documentation system (e.g., comorbidities).

All evaluations performed in this study refer exclusively to complete data sets. Consequently, some evaluations relate to specific subpopulations and are therefore not directly comparable with one another. For sake of clarity, the respective underlying dataset and corresponding subset of analyzable data are reported for each analysis.

Nevertheless, the benchmarking initiative offers substantial insights, as the standardized data collection of CDS-BARS enables comparisons across different wards and supports quality improvement at the structural, process, or outcome level. It allows participating units to benchmark their performance against comparable wards and to learn from best-practice examples [22], promotes compliance of healthcare professionals [23], and facilitates data visualization, thereby providing an objective basis for individual performance.

## Conclusion

The results of this study provide data-based insights into Austria’s acute geriatric wards and illustrate the role and potential of inpatient acute geriatric wards for the treatment of older patients with multiple morbidities in Austria. The analysis of systematically collected and standardized data in a benchmarking and reporting system like the CDS-BARS enhances transparency with regard to treatment outcomes. Improvements in self-care ability and mobility, the high proportion of patients discharged to their home environment, and the broad spectrum of admission diagnoses and comorbidities undercore the clinical relevance of inpatient acute geriatric wards. The close collaboration between the Austrian Society for Geriatrics and Gerontology (ÖGGG), the Association for Quality in Geriatrics and Gerontology (QiGG), and JOANNEUM RESEARCH provides a robust data basis for the evidence-based planning and future development of geriatric care structures in Austria.

In light of demographic change and the increasing demand for geriatric care, the ongoing development and strengthening of acute geriatric wards in Austria represent a health policy priority.
